# A Direct Interaction with NEDD1 Regulates γ-Tubulin Recruitment to the Centrosome

**DOI:** 10.1371/journal.pone.0009618

**Published:** 2010-03-10

**Authors:** Jantina A. Manning, Sonia Shalini, Joanna M. Risk, Catherine L. Day, Sharad Kumar

**Affiliations:** 1 Centre for Cancer Biology, SA Pathology, Adelaide, South Australia, Australia; 2 Department of Medicine, University of Adelaide, Adelaide, South Australia, Australia; 3 Biochemistry Department, University of Otago, Dunedin, New Zealand; University of Birmingham, United Kingdom

## Abstract

The centrosome is the primary microtubule organizing centre of the cell. γ-tubulin is a core component of the centrosome and is required for microtubule nucleation and centrosome function. The recruitment of γ-tubulin to centrosomes is mediated by its interaction with NEDD1, a WD40-repeat containing protein. Here we demonstrate that NEDD1 is likely to be oligomeric *in vivo* and binds directly to γ-tubulin through a small region of just 62 residues at the carboxyl-terminus of the protein. This carboxyl-terminal domain that binds γ-tubulin has a helical structure and is a stable tetramer in solution. Mutation of residues in NEDD1 that disrupt binding to γ-tubulin result in a mis-localization of γ-tubulin away from the centrosome. Hence, this study defines the binding site on NEDD1 that is required for its interaction with γ-tubulin, and shows that this interaction is required for the correct localization of γ-tubulin.

## Introduction

Microtubule nucleation is crucial for the establishment of a bipolar mitotic spindle and the correct division of a cell into two daughter cells with an exact set of chromosomes. Organization of microtubules is also important for other processes throughout the cell cycle such as establishing and maintaining cell polarity and shape, and transporting proteins, vesicles and organelles within the cell [1], [2]. The γTuRC (γ-tubulin ring complex) is the main effecter of this process and consists of γ-tubulin, GCPs (γ-tubulin complex proteins) and NEDD1 (neural precursor cell expressed developmentally down-regulated gene-1) [3], [4], [5]. Whilst the GCPs are involved in assembly of the γTuRC, the primary function of NEDD1 appears to be the recruitment of this complex to centrosomes and the mitotic spindle, to promote the polymerization of centrosomal microtubules in interphase and spindle microtubules in mitosis [5], [6], [7], [8].

Although many other proteins contribute to the recruitment of the γTuRC to sites of microtubule nucleation, they often exert their effects by controlling the localization or phosphorylation of NEDD1. In particular, NEDD1 is phosphorylated by Cdk1 which leads to the recruitment of Plk1 and further phosphorylation of NEDD1 [9]. These modifications promote the interaction of NEDD1 with γ-tubulin and are critical for targeting γ-tubulin to the centrosome. Other proteins that have also been shown to be important for targeting NEDD1 and γ-tubulin to the centrosome include Cdk5Rap2 and Cep72 [10], [11], as well as Cep192 and pericentrin [12], [13]. Additionally, the spindle associated protein FAM29A interacts with NEDD1 and is required for localization of NEDD1 and γ-tubulin to the mitotic spindle [14], [15]. These studies suggest that the interaction of γ-tubulin with NEDD1 is critical for its localization and function.

Previous studies have shown that the 89 amino acids at the C-terminus of NEDD1 (residues 572–660) interact with the γTuRC [7], [8], but it is not known if this interaction is direct or requires additional proteins. In this study, we report that NEDD1 interacts directly with γ-tubulin, and requires no additional binding partners. Whilst it is likely that full length NEDD1 is oligomeric *in vivo*, the 62 residues at the C-terminus (599–660), which form a helical tetramer in solution, are necessary and sufficient for the interaction with γ-tubulin. Over-expression of just these residues disrupts recruitment of γ-tubulin to the centrosome because this region of NEDD1 binds to γ-tubulin and keeps it away from the centrosome, thereby acting as a dominant negative. Importantly, we identify specific residues that are required for the NEDD1/γ-tubulin interaction, but do not compromise the tetrameric conformation of the C-terminus of NEDD1. Mutation of these residues reverses the dominant negative effect of this C-terminal fragment of NEDD1, suggesting that they are critical for its interaction with γ-tubulin.

## Results

### Residues 572–660 at the C-Terminus of NEDD1 Interact with γ-Tubulin Directly

**Figure 1:**
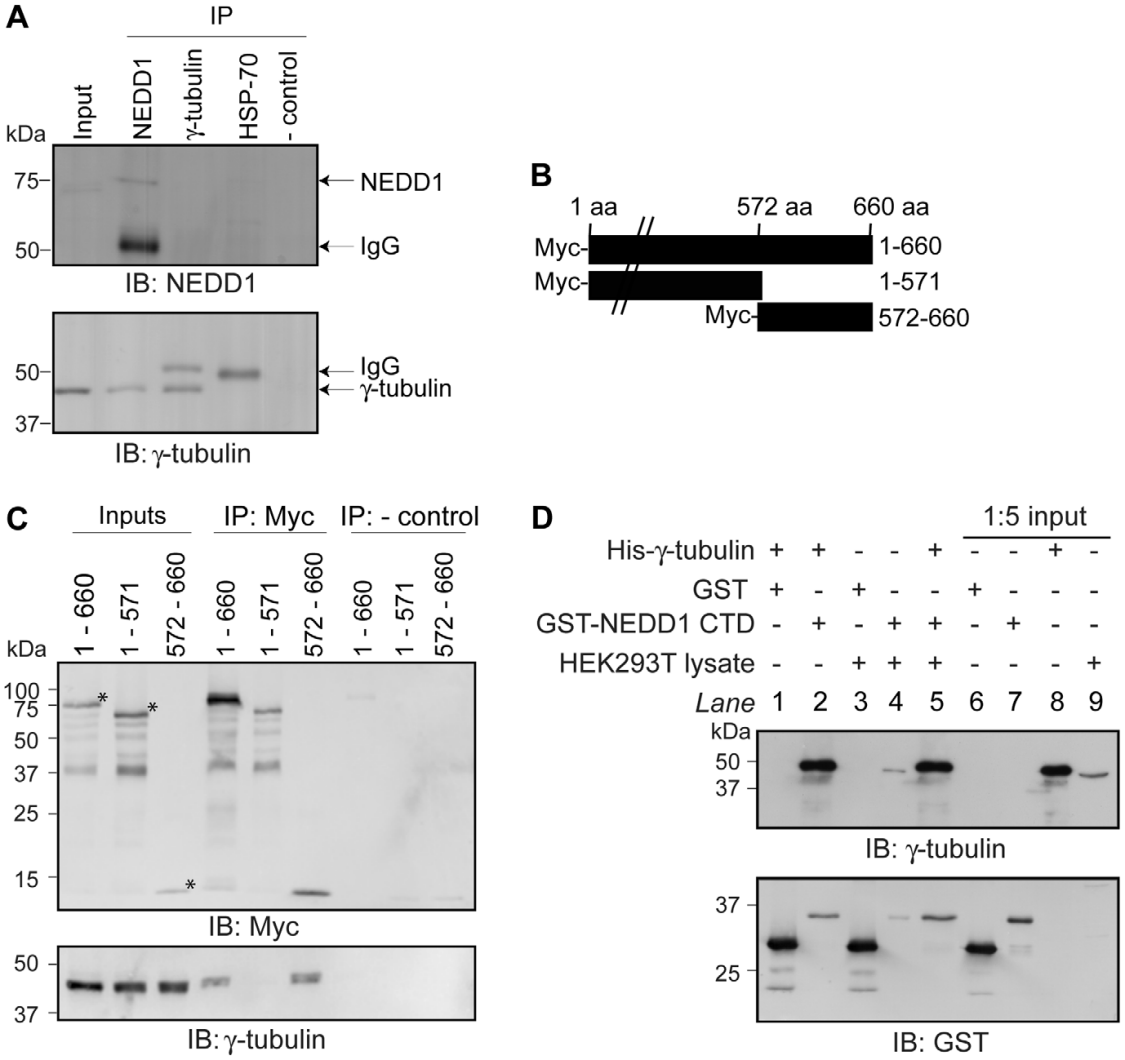
NEDD1 residues 572–660 interacts with γ-tubulin directly. (A) The interaction of endogenous NEDD1 and γ-tubulin was assessed in HEK293T cells. Due to its low level of expression, NEDD1 is not detectable in the inputs (1/20 lysates loaded), however γ-tubulin is present (first lane). Both NEDD1 and γ-tubulin are detected in lysates immunoprecipitated with NEDD1 antibody (second lane). NEDD1 is not detected when γ-tubulin is immunoprecipitated (third lane). Additional bands are IgG. In negative controls, γ-tubulin and NEDD1 are not detected when an unrelated antibody (HSP70) (fourth lane) or no antibody (fifth lane) was used for the immunoprecipitation. (B) Full length NEDD1 (660 aa), or two truncation constructs (1–571 and 572–660) were fused to a Myc-tag at their N-terminus. (C) The interaction of full length and truncated forms of Myc-NEDD1 with endogenous γ-tubulin was assessed in HEK293Ts. Expression is confirmed in the inputs (1/20 lysates loaded). γ-tubulin is immunoprecipitated with full length (660 aa) NEDD1, and 572–660 NEDD1, but not 1–571, using a Myc antibody. * represent the correct size for each construct. When no antibody is added (negative controls), no γ-tubulin is immunoprecipitated. (D) The interaction of NEDD1 CTD (residues 572–660) and γ-tubulin was assessed *in vitro*. Recombinant GST or GST-NEDD1 CTD bound to glutathione sepharose beads were incubated with His-γ-tubulin, with or without HEK293T lysate. Inputs (1/5 lysates loaded) are shown in lanes 6–8. Endogenous γ-tubulin is expressed in the lysate (lane 9). After incubation with the beads and removal of unbound proteins, γ-tubulin is not bound to GST alone (lane 1), but is bound to GST-NEDD1 CTD both in the absence and presence of lysate (lanes 2 and 5 respectively). Endogenous γ-tubulin in the lysate does not to bind to GST alone (lane 3), but does bind to GST-NEDD1 CTD (lane 4).

Previous studies have shown that NEDD1 interacts with γ-tubulin [7], [8]. We first confirmed this interaction with endogenous proteins by immunoprecipitating NEDD1 from mammalian cells, and detecting bound γ-tubulin (Figure 1A). The reciprocal immunoprecipitation of γ-tubulin did not result in any interacting NEDD1 visible on immunoblot, but this could be due to the low levels of endogenous NEDD1. As others have reported, the C-terminal residues of NEDD1 are required for this interaction and endogenous γ-tubulin is only immunoprecipitated by Myc-tagged NEDD1 constructs that contain residues 572–660 (Figure 1B and C).

We next assessed whether the interaction between NEDD1 and γ-tubulin occurs directly or is mediated by other proteins. The GST-tagged carboxyl-terminal domain (CTD) of NEDD1 (residues 572–660), and His-tagged γ-tubulin were expressed in *E. coli.* Both purified proteins were added together to glutathione sepharose beads either with, or without a mammalian cell lysate that would contain any additional binding partners. We found that γ-tubulin was bound to NEDD1 in the presence, or absence, of HEK293T lysate (Figure 1D). This indicates that NEDD1 CTD can bind to γ-tubulin directly.

### Full Length NEDD1 Is Oligomeric, with the CTD Forming a Helical Tetrameric Structure

**Figure 2:**
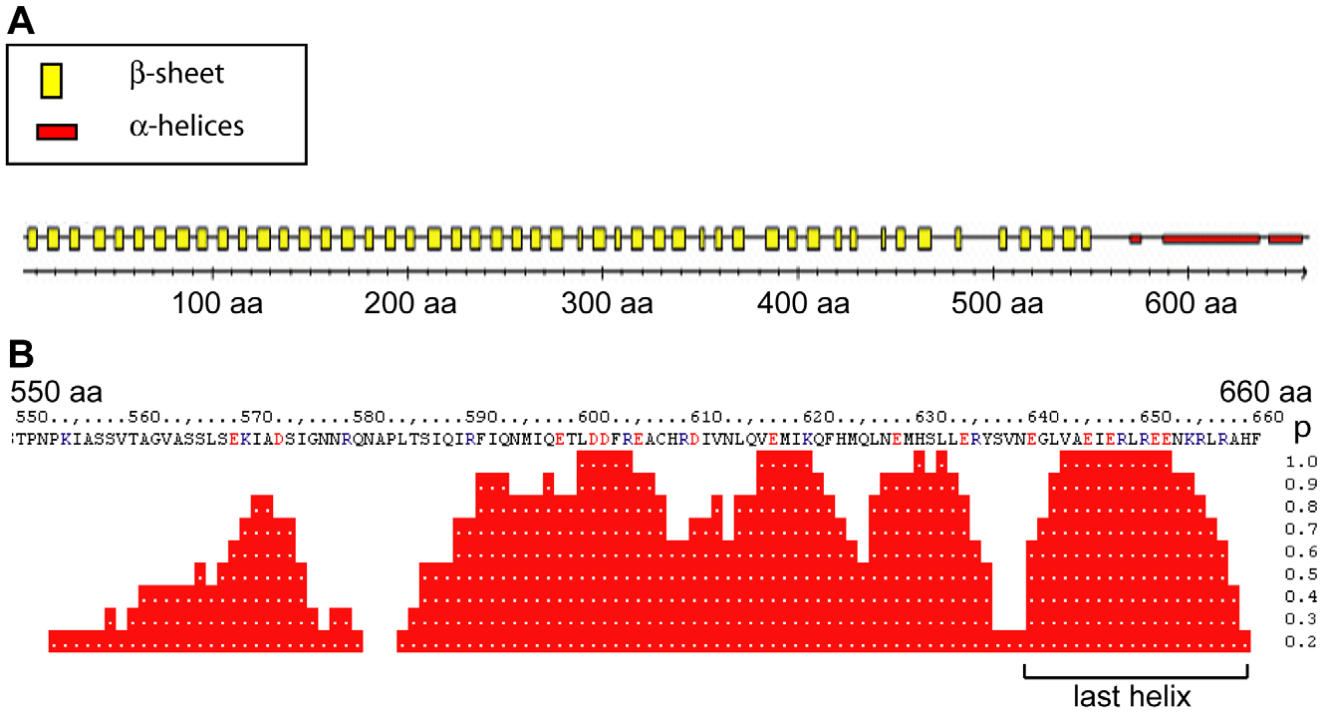
NEDD1 interacts with γ-tubulin through a helical region. PredictProtein (www.predictprotein.org) was used to assess the secondary structure of human NEDD1. (A) The majority of NEDD1 protein is predicted to be composed of β-sheets, spanning amino acids 1–550. However, the region of NEDD1 between amino acids 550–660 is predicted to be predominantly α-helical. (B) Closer analysis of the α-helical structure for residues 550–660 reveals that this region is predicted to encode three regions with a high probability (p) of being helical. The last helix (640–660 aa) has a particularly high probability (p) of being helical.

**Figure 3:**
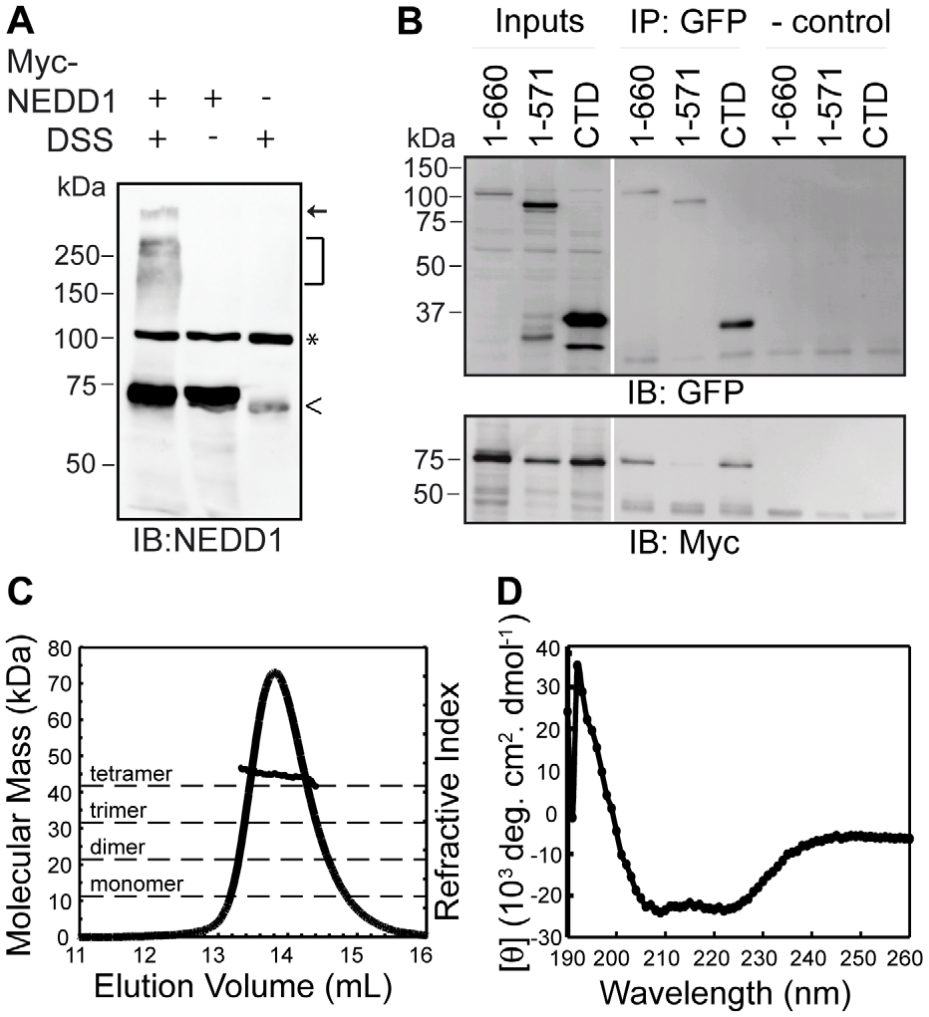
NEDD1 CTD can interact with itself and forms a helical tetramer. (A) Full length Myc-NEDD1 transfected HEK293T cells were incubated with and without DSS crosslinker, subjected to reducing SDS-PAGE and blotted for NEDD1. As well as the monomeric form of NEDD1 (∼71 kDa), DSS crosslinking allows detection of NEDD1 in higher molecular mass complexes that are not present without the crosslinker or in untransfected cells. Arrow represents high molecular mass aggregate at top of resolving gel. Bracket represents high molecular mass complexes containing NEDD1. ^ represents endogenous NEDD1. * represents a non-specific band detected by the NEDD1 antibody. (B) Full length Myc-NEDD1 and either full length (1–660), or the WD40 repeats (1–571) or residues 572–660 (CTD) GFP-NEDD1 were transfected into HEK293T cells. The expression of all NEDD1 constructs is confirmed in the inputs (Inputs, 1/20 lysates loaded). Full-length Myc-NEDD1 is immunoprecipitated with full length (1–660), and GFP-NEDD CTD, but not 1–571 GFP-NEDD1. In the absence of added antibody no NEDD1 is immunoprecipitated (- control). (C) Analysis of the oligomeric state of NEDD1 CTD by multiple angle light scattering (MALS). The predicted molecular mass of this protein is 10.5 kDa. The measured molecular mass from the MALS is 40.16 kDa suggesting that the protein is tetrameric in solution. (D) Analysis of NEDD1 CTD secondary structure by circular dichroism (CD) spectroscopy suggests that this protein is predominantly α-helical (i.e. spectra has minima at 208 and 222 nm).

Sequence analysis suggested that the NEDD1 CTD that binds to γ-tubulin has a helical structure, with the most C-terminal region having the highest probability of forming a helix (Figure 2A and B). Additionally, the NEDD1 CTD is predicted to have a coiled-coil structure (data not shown), therefore we next assessed whether this protein is multimeric in cells. To do this, full-length Myc-NEDD1 was transfected into cells and the lysate treated with the chemical crosslinker disuccinimidyl suberate (DSS), to form irreversible amide bonds between interacting proteins. As well as detecting monomeric NEDD1 at the expected mass of ∼71 kDa, DSS treatment revealed the presence of NEDD1 in a higher molecular mass aggregate at the top of the resolving gel. NEDD1 was also present in complexes ranging from ∼150–300 kDa. These complexes were not detected after reducing the sample when no DSS was present (Figure 3A). To assess whether these complexes may contain NEDD1 oligomers, we next tested whether NEDD1 could self-associate. Indeed, either GFP-fused full length NEDD1 (residues 1–660) or the CTD could immunoprecipitate Myc-tagged full length NEDD1, but residues 1–571 of NEDD1 could not (Figure 3B). This indicates that NEDD1 can interact with itself, and the CTD is required for this self-association to occur.

To further investigate the nature of the oligomer formed we analysed NEDD1 CTD by multiple angle light scattering (MALS) coupled to size exclusion chromatography (SEC), which allows the determination of absolute mass independent of shape. Using this technique we obtained a weight averaged molecular mass of 40.16 kDa for the CTD of NEDD1 (Figure 3C). The calculated molecular mass of this domain is 10.5 kDa suggesting that the CTD forms tetramers in solution. An additional fragment of NEDD1 that included residues 549–660 was also analysed by SEC-MALS (data not shown). This protein has a predicted mass of 12.8 kDa and we observed a single peak that was not affected by dilution, and had a weight averaged molecular mass of 48.5 kDa. Together these data suggest that the CTD of NEDD1 forms a stable tetramer in solution. Consistent with predictions that suggest this region of NEDD1 is likely to have a coiled-coil structure, circular dichroism spectroscopy revealed that NEDD1 CTD is predominantly α-helical (Figure 3D). Thus our results indicate that the CTD of NEDD1 forms a tetrameric helical coiled-coil.

### Residues 599–660 of NEDD1 Interact with γ-Tubulin

**Figure 4:**
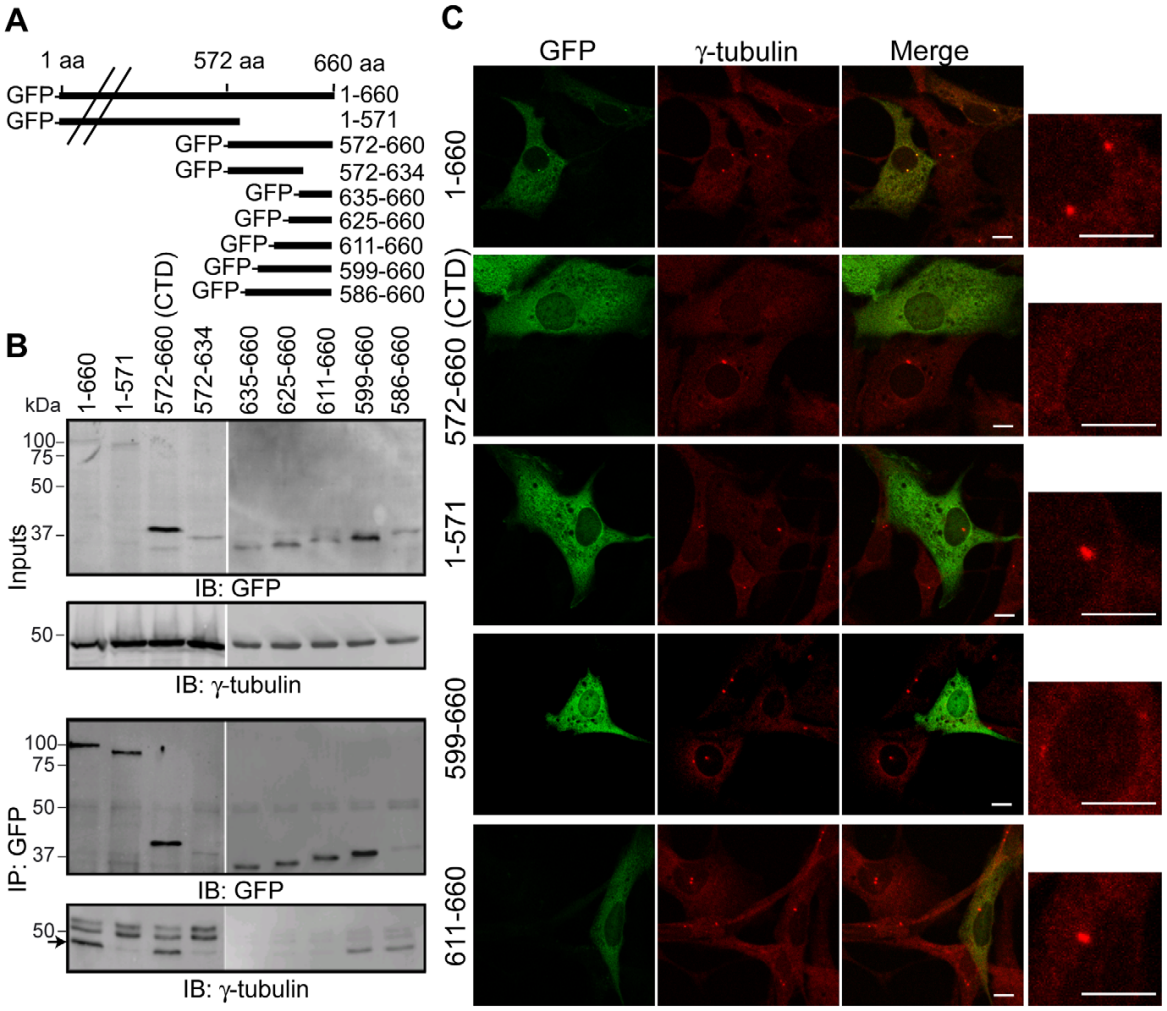
Residues 599–660 of NEDD1 interact with γ-tubulin and prevent it from localizing to the centrosome. (A) GFP-tagged NEDD1 expression constructs used in the study. (B) Interaction of NEDD1 and γ-tubulin was assessed with endogenous γ-tubulin and GFP-tagged NEDD1 expression constructs in HEK293T cells. The expression of γ-tubulin and all NEDD1 constructs is confirmed in the inputs (Inputs, 1/20 lysates loaded). All GFP-NEDD1 constructs can be immunoprecipitated with a GFP antibody (IP: GFP). However, γ-tubulin (arrow) is only immunoprecipitated with full length (1–660), 572–660 (CTD), 586–660 and 599–660 NEDD1. The upper bands in the γ-tubulin immunoblot are IgG. (C) NIH-3T3 cells were transfected with various GFP-NEDD1 constructs and immunostained for GFP (green) and γ-tubulin (red). Each image contains representative transfected and non-transfected cells. The box on the right is an enlargement of the γ-tubulin stained centrosomes in the transfected cells. Full length GFP-NEDD1 (1–660 aa) localizes to the centrosome and does not alter γ-tubulin levels at the centrosome. No other GFP-NEDD1 fusion proteins are detected at the centrosome. GFP-NEDD1 CTD and 599–660 prevent γ-tubulin from localizing to the centrosome, whereas GFP-NEDD1 (1–571) has no affect on γ-tubulin at the centrosome. All other constructs which are not able to bind to γ-tubulin have no effect (611–660 shown as an example). Scale bars  = 10 µm.

To determine where γ-tubulin bound within this helical domain, a series of NEDD1 deletion constructs were generated (Figure 4A). As sequence analysis suggested that the helical structure was disrupted at residue 635 (Figure 2B), we initially investigated if either residues 635–660, or residues 572–634 were sufficient for binding to γ-tubulin. As shown previously, when immunoprecipitated with a GFP antibody, full length NEDD1 and the CTD were able to co-precipitate γ-tubulin, but the construct that terminated at residue 571 did not (Figure 4B). However, γ-tubulin was not co-precipitated by either the N- (residues 572–634) or C- (residues 635–660) terminal fragment, suggesting that neither region alone was sufficient for binding. Using an additional series of deletion constructs γ-tubulin was found to bind to a protein that encompassed residues 599–660, but not to shorter proteins.

### Residues 599–660 of NEDD1 Prevent γ-Tubulin from Localizing to the Centrosome

Previously it has been shown that the C-terminal half of NEDD1 does not localize to the centrosome, but over-expression of this truncated protein causes a loss of γ-tubulin from the centrosome by keeping it in the cytoplasm [8]. To investigate if residues 599–660 of NEDD1 were sufficient for binding γ-tubulin *in vivo*, we tested whether this region could also interfere with γ-tubulin localization to the centrosome. As expected, moderate levels of GFP-tagged full length NEDD1 localized to the centrosome, as well as the cytoplasm, and did not have any effect on the amount of γ-tubulin at the centrosome when compared to untransfected cells (Figure 4C). In contrast, expression of GFP-NEDD1 CTD that binds to γ-tubulin, did not localize to the centrosome and resulted in a dramatic reduction of γ-tubulin levels at the centrosome. Expression of GFP-NEDD1 (residues 1–571) that is unable to bind γ-tubulin and also does not localize to the centrosome, did not alter the levels of γ-tubulin at the centrosome. Importantly, GFP-NEDD1 (residues 599–660), which was the minimal region found to interact with γ-tubulin, also resulted in a reduction of γ-tubulin levels at the centrosome. In contrast, γ-tubulin levels at the centrosome were not affected by expression of constructs that did not co-immunoprecipitate γ-tubulin (residues 611–660 shown as an example). Together these experiments suggest that residues 599–660 of NEDD1 are sufficient for the interaction with γ-tubulin and can prevent it from localizing to the centrosome, thereby acting as a dominant-negative form of NEDD1.

### Mutations in the Helical Region of NEDD1 Disrupt γ-Tubulin Binding but Not the Tetrameric Structure

**Figure 5:**
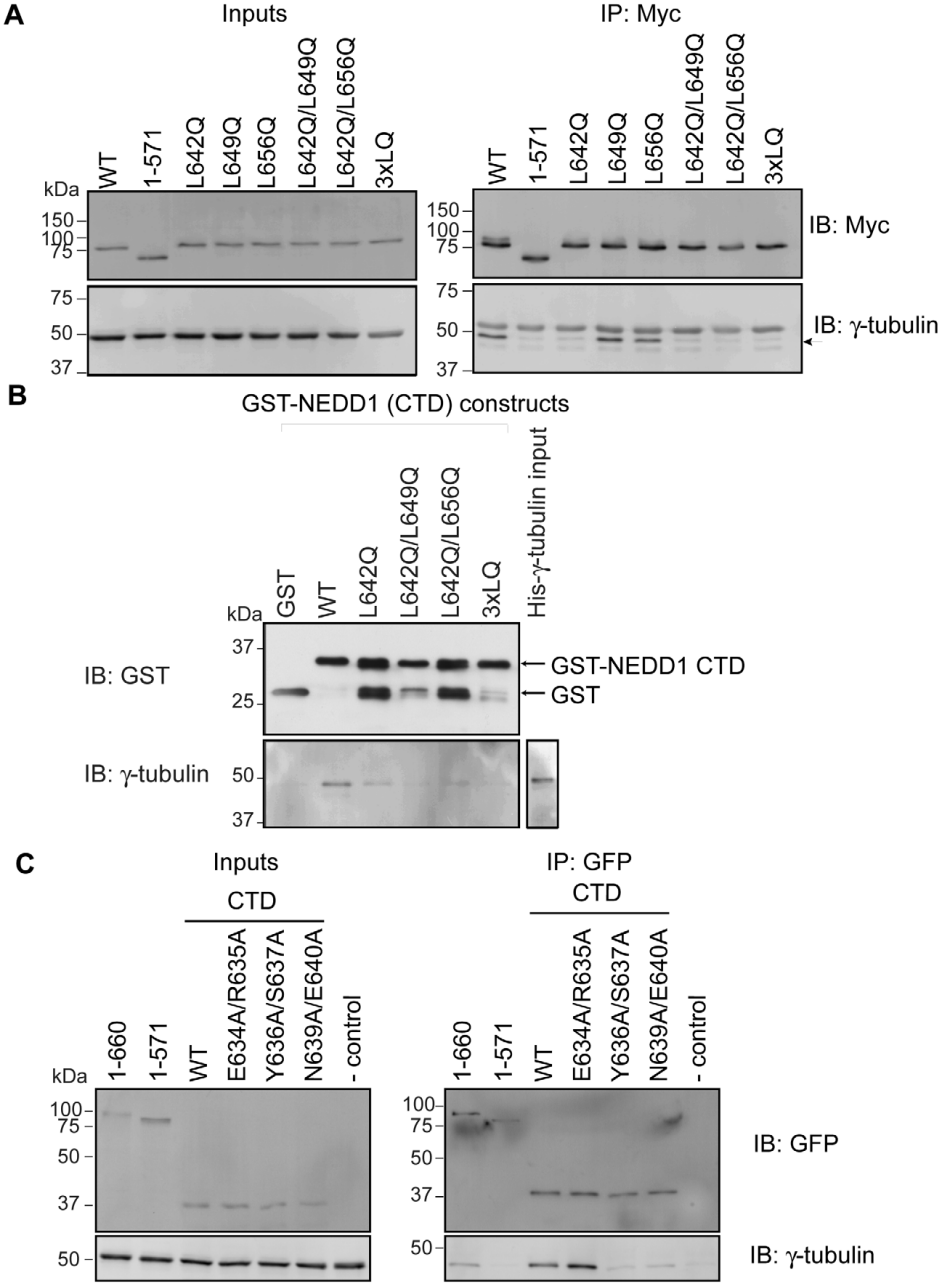
Specific mutations within NEDD1 show reduced binding to γ-tubulin. (A) Mutations were introduced into a Myc-tagged full length NEDD1 construct and transfected into HEK293T cells. The expression of all NEDD1 constructs and γ-tubulin is confirmed in the inputs (1/20 lysates loaded). γ-tubulin is immunoprecipitated with wild type (WT) full length NEDD1 but not with NEDD1 (1–571 aa). There is a loss of γ-tubulin immunoprecipitated with the L642Q mutant NEDD1, but not with the single NEDD1 mutants of L649Q or L656Q. Double mutants including L642Q also reduce the immunoprecipitation of γ-tubulin, as does the L642Q/L649Q/L656Q (3xLQ) triple mutant. The upper band in the γ-tubulin immunoblot is IgG. (B) The interaction of WT or mutant NEDD1 CTD with γ-tubulin was assessed *in vitro*. Recombinant GST or GST-NEDD1 CTD mutants bound to glutathione sepharose beads were incubated with His-γ-tubulin. All GST-tagged proteins are expressed well and bind to the beads, and His-γ-tubulin is also expressed. After incubation with the beads and removal of unbound proteins, γ-tubulin is not bound to GST alone, but is bound to WT GST-NEDD1 CTD. There is reduced binding of γ-tubulin to L642Q NEDD1, and to the double and triple mutants. The lower bands in the GST-NEDD1 CTD mutant protein lanes are likely to represent cleaved GST. (C) Selected mutations were introduced into a GFP-tagged NEDD1 CTD construct and transfected into HEK293T cells. Expression of all NEDD1 constructs and γ-tubulin is confirmed in the inputs (1/20 lysates loaded). γ-tubulin is immunoprecipitated with full length (1–660 aa) NEDD1 but not with NEDD1 (1–571 aa). WT NEDD1 CTD is able to immunoprecipitate γ-tubulin, as seen previously, as is the E634A/R635A mutant. There is a loss of γ-tubulin immunoprecipitated with the Y636A/S637A and N639A/E640A mutant constructs of NEDD1. The lane indicating - control has no antibody added.

To further define the region of NEDD1 required for interaction with γ-tubulin, we initially converted three Leu residues (642, 649 and 656) to Gln. These mutations were introduced into full-length Myc-NEDD1 either alone or in combination (Figure 5A). As before, immunoprecipitation experiments were used to assess binding. The NEDD1 L642Q mutation resulted in a dramatic loss of γ-tubulin binding, whereas the other mutations, L649Q and L656Q, appeared to have no effect (Figure 5A). As expected, double and triple mutants (3xLQ) containing the L642Q mutation also had a reduced ability to bind γ-tubulin. To further study the effects of the mutations on γ-tubulin binding, we also generated these mutants in the CTD of NEDD1 that was GST-tagged. As before, pulldown assays showed that His-tagged γ-tubulin was able to directly bind to wild type GST-NEDD1 CTD protein but the interaction of γ-tubulin with the L642Q mutant was significantly reduced (Figure 5B). The double mutants and triple mutants also had reduced binding to γ-tubulin.

### Additional Mutants of NEDD1 also Contribute to the Binding Site for γ-Tubulin

Sequence analysis of NEDD1 suggests that residues 635–642 have a reduced propensity to form a helix (Figure 2B) and analysis of the CD data at 222 nm predicts that the purified CTD protein is only 70% helical ([θ]_222_ value of -35 000 degrees cm^2^ dmol^−1^ was taken to correspond to 100% helix, see methods). Because our previous data suggested that Leu^642^ is involved in γ-tubulin recruitment, we hypothesized that the adjacent residues may not form part of a helical structure and might also contribute to the binding site. Therefore, to further dissect the requirements for binding, additional surrounding residues were mutated. Mutation of Glu^634^/Arg^635^ of NEDD1 to Ala had no effect on the binding of GFP-NEDD1 CTD to γ-tubulin, however mutation of Tyr^636^/Ser^637^, and Asn^639^/Glu^640^ caused a dramatic loss of binding, and the NEDD1 CTD proteins that contained these mutations could no longer co-immunoprecipitate γ-tubulin (Figure 5C).

**Figure 6:**
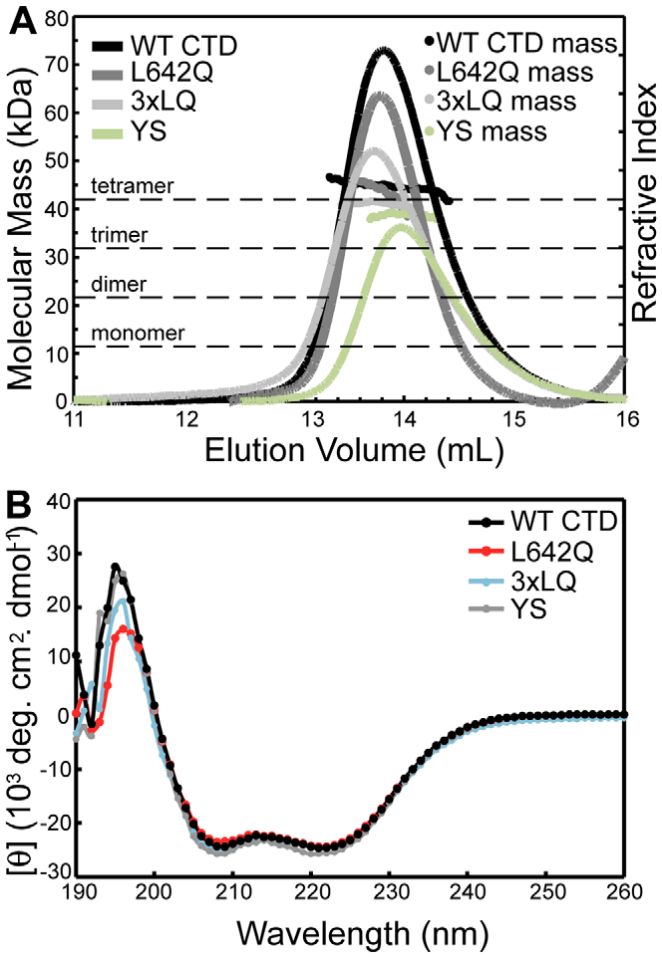
NEDD1 L642Q mutant exists as a helical tetramer. (A) Analysis of the oligomeric state of NEDD1 CTD WT, L642Q, triple 3xLQ and the YS (Y636A/S637A) mutant NEDD1 by MALS. The predicted molecular mass of these truncated constructs is 10.5 kDa. The MALS data shows a molecular mass of approx 40 kDa for each construct suggesting that the WT and the mutant proteins are tetrameric in solution. (B) Analysis of NEDD1 CTD WT and the mutants by CD spectroscopy suggests these proteins are predominantly α-helical (spectra has minima at 208 and 222 nm).

Since it was possible that mutations had disrupted tetramer formation and that changes in the overall structure might account for the reduced binding of γ-tubulin, we next analysed purified CTD containing these mutations by CD and MALS. This analysis showed that the L642Q and triple 3×LQ mutants in the NEDD1 CTD were still tetrameric, as like wild type protein, they had an average molecular mass of approximately 40.16 kDa (Figure 6A), and were helical in solution (Figure 6B). The additional mutants within residues 634–640 were also still found to be tetrameric and helical, (shown with Y636A/S637A mutant, Figure 6A and B), suggesting that these residues contribute to the interaction interface rather than playing a structural role. Together this suggests that residues 636–642 of NEDD1 have a critical role in recruitment of γ-tubulin.

### Mutations of NEDD1 within the Helical Structure Affect γ-Tubulin Localization

**Figure 7:**
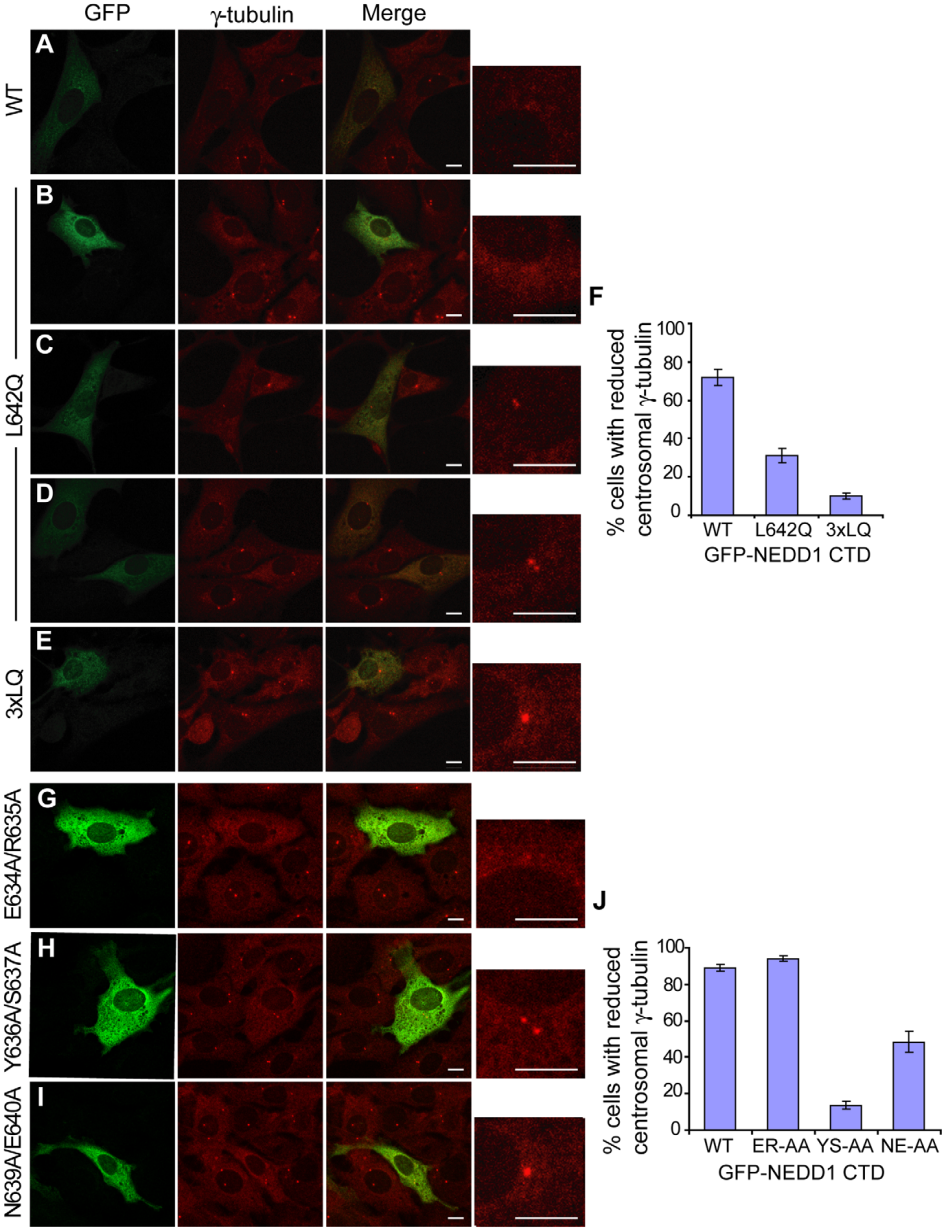
Specific NEDD1 mutations with reduced binding to γ-tubulin also prevent it from localizing to the centrosomes. (A-E, G-I) The localization of WT and mutant GFP-NEDD1 CTD protein and their effects on γ-tubulin were assessed in NIH-3T3 cells. Cells are stained with GFP (green) and γ-tubulin (red). Each image contains representative transfected and non-transfected cells. The box on the right is an enlargement of the γ-tubulin stained centrosomes in the transfected cells. Scale bars  = 10 µm. (A) WT NEDD1 CTD does not localize to the centrosome, and causes a dramatic reduction in γ-tubulin levels at the centrosome. *B-D.* L642Q NEDD1 CTD does not localize to the centrosome, and has varied effects on the centrosomal localization of γ-tubulin. In some cells, γ-tubulin levels are greatly reduced at the centrosome (B), whereas in most cells the level of γ-tubulin appears similar to untransfected cells [small reduction in (C), normal levels in (*D*)]. (E) The 3xLQ triple mutant NEDD1 CTD does not affect the localization of γ-tubulin to the centrosome in the majority of cells. (F) The number of cells with reduced levels of γ-tubulin at the centrosome was counted for each transfection. Error bars show SEM, where n = 4 independent experiments. (G) E634A/R635A NEDD1 CTD does not localize to the centrosome, and causes a dramatic reduction in γ-tubulin levels at the centrosome. (H) The Y636A/S637A mutant does not affect the localisation of γ-tubulin to the centrosome in the majority of cells. (I) The N639A/E640A mutant also does not affect the localisation of γ-tubulin to the centrosome in a high proportion of cells. (J) The number of cells with reduced levels of γ-tubulin at the centrosome was counted for each transfection. Error bars show SEM, where n = 3 independent experiments.

We then assessed whether the NEDD1 mutants that showed reduced binding to γ-tubulin could recruit γ-tubulin to the centrosome using the previously generated GFP-tagged NEDD1 CTD mutant proteins. As in previous experiments, this short form of NEDD1 did not localize to the centrosome, and caused a dramatic reduction of γ-tubulin at the centrosome by keeping this protein in the cytoplasm and acting as a dominant negative (Figure 7A). The L642Q mutation had varied effects on the abundance of γ-tubulin at the centrosome, depending on the expression level of the protein. In some cells, the L642Q construct appeared to behave almost identically to wild type, causing a dramatic loss of γ-tubulin from the centrosome (Figure 7B). However, in the majority of cells there was either a small reduction in the levels of γ-tubulin at the centrosome (Figure 7C), or centrosomal γ-tubulin was comparable to untransfected cells (Figure 7D) suggesting that the CTD containing the L642Q mutation was unable to prevent γ-tubulin from localizing to the centrosome. The triple 3xLQ mutant GFP-NEDD1 CTD displayed a more consistent effect, with most cells retaining strong γ-tubulin staining in the centrosome indicating that this mutant was not able to recruit γ-tubulin (Figure 7E). The numbers of cells with reduced levels of γ-tubulin at the centrosome were counted. Whilst 72% of cells transfected with GFP-NEDD1 CTD displayed reduced levels of γ-tubulin at the centrosome, only 31% of cells transfected with the L642Q mutant, and 10% of cells transfected with the triple mutant had reduced γ-tubulin at the centrosome (Figure 7F). These results suggest that the L642Q mutation compromises the binding of NEDD1 to γ-tubulin *in vivo*, and that the additional mutations in the triple L642Q/L649Q/L656Q mutant have an additive effect, although the individual mutants did not disrupt binding (Figure 5A).

We also analyzed the other CTD mutants we had generated and as expected, the E634A/R635A mutant that can still bind to γ-tubulin, decreased γ-tubulin levels at the centrosome similar to when cells were transfected with wild type CTD (compare Figure 7A and G). In contrast, mutant Y636A/S637A, which is no longer able to bind γ-tubulin, had normal levels of γ-tubulin at the centrosome (Figure 7H). The N639A/E640A construct produced more variable results, although it was still apparent that many cells displayed normal γ-tubulin localization at the centrosome, consistent with an impaired ability to bind to γ-tubulin (Figure 7I). Quantification showed that approximately 90% of cells transfected with WT and Glu^634^/Arg^635^ mutant CTD NEDD1 had reduced γ-tubulin at the centrosome, whereas only 14% of cells transfected with Tyr^636^/Ser^637^ mutant CTD NEDD1 and 48% of cells containing the Asn^639^/Glu^640^ mutant CTD NEDD1 had reduced γ-tubulin at the centrosome (Figure 7J). These experiments establish a critical role for residues 636–642 of NEDD1 in recruiting γ-tubulin to the centrosome.

Together our data is consistent with the CTD of NEDD1 having a helical structure and associating to form a coiled coil. However, the region that interacts with γ-tubulin is likely to have a more extended structure and specific residues within this region contribute to the interaction interface.

## Discussion

NEDD1 has previously been identified as a component of the γTuRC, along with the γTuRC proteins GCPs 2–6. The main function of the GCPs is in microtubule nucleation, whereas NEDD1 appears to be involved in the recruitment of the γTuRC to the centrosome [3], [7], [8]. This has been shown to occur through the interaction of NEDD1 with γ-tubulin. Because of this, loss of NEDD1 results in a failure of the γTuRC to localize to both the centrosome and the mitotic spindle, which ultimately results in spindle defects and cell cycle arrest [7], [8], [16]. Here we show that the interaction between NEDD1 and γ-tubulin is direct and does not require additional proteins such as the GCPs. This suggests that other regulators of this complex, such as the centrosomal proteins Cep192, pericentrin, Cdk5Rap2, Cep72 [10], [11], [12], [13], the mitotic spindle protein FAM29A [14], and the mitotic kinases Plk1 and Cdk1 [9], [17], lie upstream of NEDD1. Indeed, NEDD1 appears to be part of a complex. Using chemical crosslinking, full length NEDD1 was detected in a high molecular mass aggregate, likely representing NEDD1 in a complex with other proteins. Additional NEDD1-containing complexes were present between ∼150–300 kDa, including a band at ∼284 kDa potentially representing tetrameric NEDD1. Although the nature of the complexes remains speculative, NEDD1 was found to self-associate in co-immunoprecipitation experiments. Futhermore, the C-terminal region of NEDD1 (CTD) that interacts with γ-tubulin, formed a stable tetramer *in vitro* and was shown to self associate *in vivo*. Our data do not directly show if endogenous NEDD1 protein is tetrameric in cells. However, combined observations of the high molecular mass complex, association of the full length protein in transfected cells and the ability of the CTD to form a tetramer *in vitro*, suggest that NEDD1 may function as an oligomer *in vivo*.

This study also confined the region responsible for the NEDD1/γ-tubulin interaction to residues 599–660 of NEDD1. Indeed, this region of NEDD1 does not localize to the centrosome, but since it binds to γ-tubulin, is able to prevent γ-tubulin from localizing to the centrosome. In a recent study which showed that phosphorylation of NEDD1 by Cdk1 and Plk1 is important for its interaction with γ-tubulin, four Plk1 phosphorylation sites (Ser^382^, Ser^397^, Ser^426^ and Ser^637^) were identified [9]. Mutation of all of these sites in combination reduced the binding of NEDD1 and γ-tubulin, although it was not abolished [9]. Since our data show that recombinant unphosphorylated NEDD1 CTD (residues 572–660) can bind γ-tubulin, this suggests that Plk1-mediated phosphorylation is not essential but may be required for either formation of a tight complex, or for interaction of full-length NEDD1 with γ-tubulin.

Initial mutations in NEDD1 suggested that residues that are not strongly predicted to adopt an α-helical conformation might provide a binding surface for γ-tubulin. Indeed, multiple mutations within residues 636–642 of NEDD1 caused a significant reduction in binding to γ-tubulin both *in vivo* and *in vitro*. These mutants are still helical and tetrameric in solution, suggesting that the mutated residues are important for direct binding of γ-tubulin and do not abrogate binding by disrupting the structure of the CTD. The mutation of residues outside this region (E634A/R635A, L649Q and L656Q) had minimal effect on γ-tubulin binding. However, whilst specific residues within the CTD create a docking site for γ-tubulin, the entire region is required for the interaction, presumably by contributing to the conformation of the protein.

In order to test the functional importance of these mutants, the localization of γ-tubulin to the centrosome was assessed in transfected cells. Due to the presence of endogenous NEDD1 interfering with localization studies, the NEDD1 CTD construct was used for these studies, because it displays a dominant-negative effect in keeping γ-tubulin away from the centrosome, overriding any endogenous NEDD1. In these experiments, expression of wild type GFP-NEDD1 CTD resulted in a reduction of γ-tubulin at the centrosome in most cells. However, the expression of all mutant proteins that have reduced binding to γ-tubulin resulted in a high proportion of cells displaying normal levels of γ-tubulin at the centrosome. Hence, these mutants are able to abrogate the binding of NEDD1 to γ-tubulin, and therefore the centrosomal localization of γ-tubulin.

Interestingly, in the *Xenopus* oocyte extracts, *Xenopus* NEDD1 (xNEDD1) depletion does not reduce the levels of γ-tubulin at the centrosome and the majority of xNEDD1 exists in a complex that does not contain γ-tubulin [18]. The main effect of xNEDD1 depletion is a reduction in γ-tubulin localizing to microtubules. However, in a recent study from our laboratory, we have shown that zebrafish NEDD1 (zNEDD1) interacts with γ-tubulin through an equivalent short region at the C-terminus of the protein, and is required for the recruitment of γ-tubulin to the centrosome [19]. It is possible that this difference in recruitment of γ-tubulin by xNEDD1 and zNEDD1 (and mammalian NEDD1) is a result of varying conditions in experimental systems, such as the contribution of maternal deposits or the extent of protein knockdown rather than a true functional difference. However, it still remains possible that there are subtle differences in the function of NEDD1 from different species. Regardless of this, results from this study, and in zebrafish [19], demonstrate that the interaction of NEDD1 and γ-tubulin is important for the localization of γ-tubulin and normal development.

In summary, we have shown that NEDD1 interacts directly with γ-tubulin through a short helical region in its C-terminus. Self-association of NEDD1 was also observed and it will be interesting to investigate the stoichiometry of the complex between NEDD1 and γ-tubulin as the oligomeric structure of NEDD1 may be important for recruitment of γ-tubulin. In addition, it will be interesting to identify the residues on γ-tubulin that interact with NEDD1. Analysis of the structure of γ-tubulin suggests a number of potential interaction interfaces but ultimately structures of the complex will be required to reveal the molecular details of the interaction.

## Materials and Methods

### Cell Culture and Transfection

NIH-3T3s were cultured in Dulbecco's Modified Eagle's Medium (GIBCO) supplemented with 10% fetal bovine serum (FBS, GIBCO), 50 units/ml penicillin and 0.05 mg/ml streptoMycin at 37°C in 5% CO_2_. HEK293Ts were cultured in RPMI-1640 (GIBCO) with the same supplements. 24 h prior to transfection, cells were seeded in 6-well plates at 5×10^5^ cells per well for NIH-3T3s or 8×10^5^ cells per well for HEK293Ts. For cell staining, cells were seeded onto glass coverslips. Cells were transfected with 2 µg each DNA construct using Lipofectamine2000 (Invitrogen), according to instructions supplied by the manufacturer.

### Generation of Constructs

Human NEDD1 cDNA was cloned into pCMV-Myc (Invitrogen) or pDEST-53 (GFP-tag, Invitrogen). Generation of various truncated forms of NEDD1 was conducted using primers designed against specific portions of the gene. To generate a GST-fusion protein, the region encoding amino acids 572–660 was cloned into pGEX-4T3 (Amersham). To generate His-tagged γ-tubulin, the ORF of γ-tubulin was cloned into pET-15b (Novagen). Mutagenesis was carried out using the Quikchange Site-Directed Mutagenesis Kit (Stratagene). Primer sequences were as follows:

Generating Myc-tagged NEDD1 constructs: hmNd1BglIIf: GAAGATCTCTATGCAGG AAAACCTCAG, hND1XhoIr: CGCTCGAGTCAAAAGTGGGCCCG, hND1572BgIIf: TATG GCCGACAGCATTGG, hND1571XhoIr: CCGCTCGAGTCATATTTTTTCTGAGAG.

Generating His- and GST-tagged NEDD1/γ-tubulin constructs: gTubNdeIf: CATATGC CGAGGGAAATCATC, gTubBglIIr: ATCTTCACTGCTCCTGGG, hNd1572SalIf: GTCGACG CCGACAGCATTGG, hNd1NotIr: GGCGGCCGCTCAAAAGTGGGCCCG.

Generating GFP-tagged NEDD1 constructs (TOPO Gateway cloning): hNd1TOPOf: CA CCATGCAGGAAAACCTC, hNd1TOPO572f: CACCATGGCCGACAGCATTGG, hNd1TOPO635f: CACCATGAGATACTCAGTGAATG, hNd1TOPO586f: CACCATGTCCAT TCAAATTCG, hNd1TOPO599f: CACCATGACGTTGGATGACTT, hNd1TOPO611f: CACC ATGATTGTTAATTTGC, hNd1TOPO625f: CACCATGCAACTGAATGAAATGC, hNd1634Xho1r: CCGCTCGAGTCATTCCAGCAAAGAATGC, hNd1571Xho1r: CCGCTCGA GTCATATTTTTTCTGAGAG.

Mutagenesis of NEDD1: hNd1L642Qf: CTCAGTGAATGAAGGTCAAGTGGCTGAA ATTGAAAG, hNd1L642Qr: CTTTCAATTTCAGCCACTTGACCTTCATTCACTGAG, hNd1L649Qf: GTGGCTGAAATTGAAAGACAACGAGAAGAAAAC, hNd1L649Qr: GTTTT CTTCTCGTTGTCTTTCAATTTCAGCCAC, hNd1L656Qf: GAAGAAAACAAAAGACAAC GGGCCCACTTTTG, hNd1L656Qr: CAAAAGTGGGCCCGTTGTCTTTTGTTTTCTTC.

### Cell Staining and γ-Tubulin Quantification

Cells on glass coverslips were fixed in 100% methanol for 5 min at −20°C, washed in PBS and incubated in blocking solution (1% FBS/PBS) for 30 min. Primary antibodies in blocking solution were added for 2 h at room temperature (RT): 1∶1000 rabbit α-GFP (Ab290, Abcam) or 1∶500 mouse α-γ-tubulin (GTU-88, Sigma). Cells were then washed in PBS and secondary antibodies: 1∶1000 rabbit Alexa Fluor 488 and/or 1∶1000 mouse Alexa Fluor 568 (Molecular Probes) were added in blocking solution for 1 h at RT. Cells were washed again in PBS and stained with Hoechst 33342 (Molecular Probes) for 1 min. Coverslips were mounted in Prolong Gold Antifade reagent (Molecular Probes). Images were obtained using a confocal microscope (Radiance 2100, BioRad Laboratories) and processed as described previously [20]. Centrosomal levels of γ-tubulin were counted blind, directly under the microscope as classed as having normal γ-tubulin levels (seen in all control untransfected cells) or reduced/absent levels.

### Protein Expression and Purification

GST-fused proteins were expressed in *E. coli* BL21 Star (DE3) and purified as previously described [21]. The pooled eluate was dialysed against PBS overnight at 4°C and if required, protein was concentrated using YM-30 Centricon columns (Millipore). His-tagged γ-tubulin was expressed as above and purified using Ni-NTA and the QIAexpress kit (QIAGEN) under denaturing conditions in order to increase protein solubility. The eluates were pooled and protein refolded by dialysis in refolding buffer with a series of urea buffer for 24 h each (0.05 M Tris pH 8.0, 0.005% triton x-100, 2 mM GSH, urea [6M, 4M, 2M, 1M, 0]).

For biochemical analysis, NEDD1 amino acids 572–660 and mutants were expressed as GST-fusion proteins in *E. coli* BL21 star (DE3). Cells were induced at an optical density of 0.6 at 600 nm by addition of 0.2 mM isopropyl β-D-thiogalactopyranoside (IPTG) and incubated at 18°C overnight. A 500 mL cell pellet was harvested by centrifugation and cells were lysed by sonication in 20 mL TBS, pH 8.0. The fusion protein was then bound to glutathione resin (10 mL of 50∶50 slurry) for 45 min at 4°C with gentle mixing. The GST-tag was removed by overnight digestion with thrombin at 4°C. Soluble NEDD1 was recovered from the resin and purified by size exclusion chromatography (Superdex 200 HR 10/30 column, GE Healthcare). Fractions of high purity were pooled and concentrated using a Viva-spin 5 kDa cut-off concentrator (GE Healthcare) in the presence of 5 mM DTT.

### Immunoprecipitation and Immunoblotting

HEK293Ts were lysed and immunoprecipitated as previously described [22], using 1 µl of antibodies to NEDD1 [23], mouse α-γ-tubulin, mouse α-HSP70 (gift from R. Morimoto, Northwestern University, IL), goat α-Myc (Ab9132, Abcam) and rabbit α-GFP. Samples were separated by SDS-PAGE, transferred to PVDF membranes, blocked in 5% skim milk in PBST (0.1% Tween20/PBS) for 1–2 h, and incubated with primary antibodies overnight at 4°C. Primary antibodies used were: rabbit α-NEDD1 at 1∶200; mouse α-γ-tubulin at 1∶5000; mouse α-Myc at 1∶1000 (9E10, Roche); rabbit α-GST at 1∶2000 (serum purified from rabbits inoculated with a GST-fusion protein) [as in 23]; goat α-GFP at 1∶1000; mouse α-HSP70 at 1∶5000; mouse α-GFP at 1∶1000 (7.1/13.1, Roche). After washing in PBST, membranes were incubated for 2 h at RT with 1∶1000 secondary antibodies; Horseradish Peroxidase (HRP) conjugated (GE Healthcare) to detect NEDD1 or Alkaline Phosphatase (AP) conjugated (Chemicon) to detect all other proteins. HRP signals were detected using ECL Plus (GE Healthcare) and developed on X-ray film (Fujix). AP signals were detected using ECF (GE Healthcare) on a Typhoon 9410 (Molecular Dynamics) and analyzed by ImageQuant software (GE Healthcare).

### DSS Crosslinking

HEK293T cells transfected with 2 µg Myc-NEDD1, or untransfected cells, were lysed in modified lysis buffer containing no Tris-HCL (20 mM HEPES pH 7.0, 1% TritonX-100, 10% glycerol, 150 mM NaCl, 2 mM EDTA and complete protease inhibitors [Roche]) by three cycles of freeze/thaw in liquid nitrogen. Debris was removed by centrifugation at 16000*x*g for 2 min and the supernatants incubated with 1 mM disuccinimidyl suberate (DSS, Thermo Scientific) for 15 min on ice. 2x protein loading buffer (100 mM Tris-HCl pH 6.8, 200 mM DTT, 4% SDS, 0.2% bromophenol blue, 20% glycerol) was added to the samples and they were boiled for 2 min and subjected to SDS-PAGE as above.

### Direct Protein Interaction Studies

GST-NEDD1 was bound to 20 µl of glutathione sepharose beads (GE Healthcare). Beads were washed twice in NTEN buffer (20 mM Tris pH 7.4, 0.1 mM EDTA, 100 mM NaCl, 0.5% NP-40) then 2.5 µg of purified protein was added with, or without 50 µg HEK293T lysate that was pre-cleared with 20 µl of glutathione sepharose beads for 1 h at 4°C and incubated overnight at 4°C. Beads were washed 4x with NTEN buffer and bound proteins eluted by boiling for 5 min in 2x protein loading buffer (as above).

### CD and MALS Analysis

Concentrated protein was analyzed by multiple angle light scattering (MALS) (Wyatt Technology) when coupled to a Superdex 200 HR 10/30 column (GE Healthcare) equilibrated in 50 mM NaP, 300 mM NaCl, 1 mM DTT, pH 8.0. Data was analyzed using ASTRA (Wyatt Technology). Circular dichroism (CD) spectra were acquired using an Olis spectropolarimeter equipped with a Peltier type temperature controller (Olis, Inc.). Protein concentration was typically 0.2 mg/mL and samples were in 20 mM sodium phosphate buffer, pH 8.0, 100 mM NaCl. Spectra were recorded at 10°C in a 1 mm pathlength cuvette, using a 1 nm step resolution and 3 sec integration time. Five scans were averaged for each run and appropriate baselines were subtracted. All CD scans were converted to mean residue ellipticity (deg.cm^2^.dmol^−1^). A [θ]_222_ value of −35 000 degrees cm^2^ dmol^−1^ was taken to correspond to 100% helix [24].
